# Predicting Amyloidogenic Proteins in the Proteomes of Plants

**DOI:** 10.3390/ijms18102155

**Published:** 2017-10-16

**Authors:** Kirill S. Antonets, Anton A. Nizhnikov

**Affiliations:** 1 Laboratory for Proteomics of Supra-Organismal Systems, All-Russia Research Institute for Agricultural Microbiology, Podbelskogo sh., 3, Pushkin, St. Petersburg 196608, Russia; kirantonez@gmail.com; 2 Department of Genetics and Biotechnology, St. Petersburg State University, Universitetskaya nab., 7/9, St. Petersburg 199034, Russia

**Keywords:** amyloid, Waltz, SARP, plant, prion, seed storage protein, proteomics, compositionally biased region, amyloidogenic region

## Abstract

Amyloids are protein fibrils with characteristic spatial structure. Though amyloids were long perceived to be pathogens that cause dozens of incurable pathologies in humans and mammals, it is currently clear that amyloids also represent a functionally important form of protein structure implicated in a variety of biological processes in organisms ranging from archaea and bacteria to fungi and animals. Despite their social significance, plants remain the most poorly studied group of organisms in the field of amyloid biology. To date, amyloid properties have only been demonstrated in vitro or in heterologous systems for a small number of plant proteins. Here, for the first time, we performed a comprehensive analysis of the distribution of potentially amyloidogenic proteins in the proteomes of approximately 70 species of land plants using the Waltz and SARP (Sequence Analysis based on the Ranking of Probabilities) bioinformatic algorithms. We analyzed more than 2.9 million protein sequences and found that potentially amyloidogenic proteins are abundant in plant proteomes. We found that such proteins are overrepresented among membrane as well as DNA- and RNA-binding proteins of plants. Moreover, seed storage and defense proteins of most plant species are rich in amyloidogenic regions. Taken together, our data demonstrate the diversity of potentially amyloidogenic proteins in plant proteomes and suggest biological processes where formation of amyloids might be functionally important.

## 1. Introduction

Amyloids represent protein fibrils consisting of monomers that form intermolecular β-sheets located along the axis of a fibril and are stabilized by numerous hydrogen bonds. Such a spatial structure is called “cross-β” [1]. The term “cross-β” refers to the common pattern of amyloids in X-ray diffraction analysis with two scattering signals of approximately 4.7 and 10 Å corresponding to the distances between β-strands comprising β-sheets and between intermolecular β-sheets, respectively [2,3]. Their highly ordered structure gives amyloids unusual properties including resistance to treatment with ionic detergents [4], other protein denaturants [5] and proteinases [6].

Initially, amyloids were described as lethal pathogens causing incurable diseases (amyloidoses) of humans and animals [7]. The term “amyloid” was proposed in 1854 by Rudolf Virchow, who was the first to stain pathological amyloid deposits in human tissues with iodine [8]. Though “amyloid” is a derivative from “amylon” and “amylum” (starch-like in Greek and Latin, respectively), the key components of amyloid deposits are protein fibrils [7,9]. Nevertheless, such deposits additionally contain a significant number of proteoglycans and glycosaminoglycans that were initially detected by iodine and led to an incorrect interpretation of the chemical nature of amyloids [10]. Amyloidoses occur primarily due to mutations that change the structure of the corresponding amyloid-forming proteins or lead to their overproduction [11]. To date, more than 30 human proteins have been shown to adopt pathological amyloid states [12].

Another aspect of these proteins was revealed over the last two decades, when amyloids that were not associated with pathogenesis were found. These amyloids, which are formed under native conditions and are implicated in cellular processes, were named “functional amyloids” [13,14]. In bacteria, functional amyloids are important for biofilm formation [15], toxin metabolism [16], and overcoming surface tension by aerial hyphae [17]. In archaea, such amyloids not only participate in the formation of biofilms [18] but also act as the structural components of the cell sheaths [19]. Functional amyloids of fungi regulate heterokaryon incompatibility [20] as well as facultative multicellularity [21] and, similar to bacterial amyloids, contribute to the formation of aerial hyphae [22]. Amyloids forming under native conditions in animals (including humans) are involved in long-term memory formation [23,24], melanin polymerization [25], hormone storage [26], tooth enamel polymerization, programmed necrosis [27], and antiviral responses [28]. Taken together, amyloids represent not only pathogenic but also widespread functionally important variants of the quaternary protein structure and are vital for many species.

The propensity of a protein to form amyloid fibrils is determined by the presence in its amino acid sequence of so-called “amyloidogenic regions” (ARs) that drive amyloidogenesis [29,30,31] acting as a “trigger” for polymerization [32]. Amyloid-forming proteins may contain one or multiple ARs [33,34], which are relatively short [35] and predominantly composed of hydrophobic residues, especially aromatics (W, F, Y) and aliphatics (V, I, L) [36]. ARs can be predicted using a wide range of algorithms, one of the most efficient of which is Waltz [37], which is based on a position-specific scoring matrix [36,37]. Another type of AR is represented by compositionally biased regions (CBRs) that are rich in glutamine (Q) and/or asparagine (N) [38]. The key role of QN-rich CBRs in amyloid formation was initially demonstrated on the human poly-Q expanded Huntingtin protein [39] and further deepened by the data obtained on the yeast amyloid-forming proteins [40]. In addition to QN, CBRs rich in E are also amyloid-prone [41]. Compositionally biased regions rich in Q, N or E can be efficiently predicted by different existing bioinformatic algorithms, including LPS (Lower Probability Subsequences) [42] and SARP (Sequence Analysis based on the Ranking of Probabilities) [43]. Hereafter, short amyloidogenic regions predicted with Waltz are referred to as ARs, while potentially amyloidogenic compositionally biased regions are referred to as CBRs. Currently, bioinformatic prediction is widely used for the detection of potentially amyloidogenic (i.e., containing amyloidogenic regions) proteins in the proteomes of different species [42,44,45] as well as for the identification of amyloidogenic regions in particular proteins to analyze their amyloid properties in vitro and in vivo [46,47,48].

Despite the fact that plants are one of the most economically important groups of organisms, they remain the least studied in the field of amyloid biology. To date, amyloid properties have been demonstrated for several plant proteins or their fragments only in vitro [49,50] or in heterologous systems in vivo [46] (for a review, see [51]). Here, we present a large-scale analysis of the distribution of potentially amyloidogenic proteins in the proteomes of land plants reported to date. We screened the proteomes of 75 species comprising more than 2.9 million proteins for the presence of amyloidogenic regions using the SARP and Waltz algorithms. We analyzed the molecular functions of potentially amyloidogenic plant proteins along with their subcellular localization and molecular process involvement. We found plant-specific groups of proteins in which amyloidogenic regions are overrepresented and discuss the analysis of amyloid properties of such proteins and their potential significance.

## 2. Results

### 2.1. Abundance of Potentially Amyloidogenic Proteins in the Proteomes of Plants

**Figure 1:**
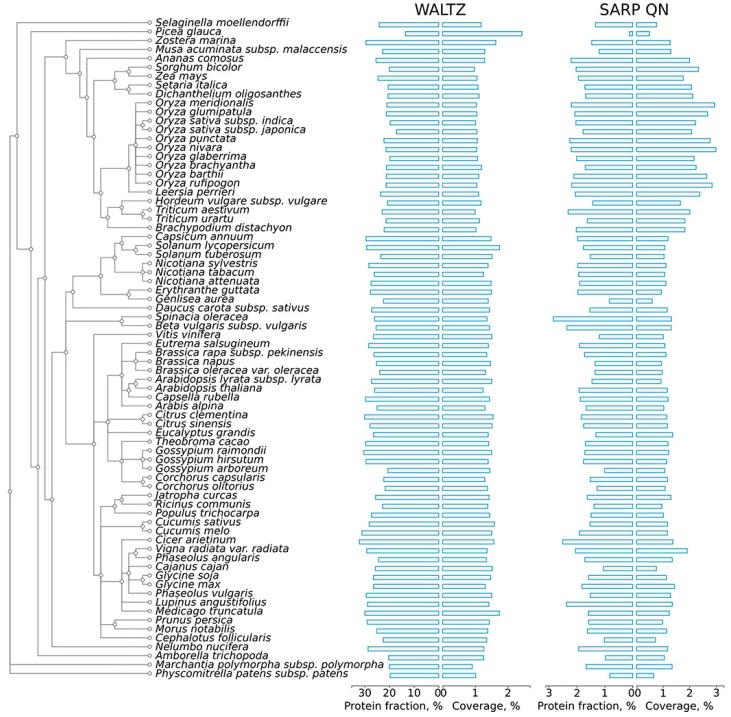
Distribution of amyloidogenic regions in the proteomes of land plants. A phylogenetic tree of plant species is shown according to the Uniprot Taxonomy. The results for proteins bearing ARs predicted by Waltz and QN-rich CBRs found with SARP are shown. For each type of amyloidogenic region, the percentage of proteins harboring these regions (%) and the coverage of the total proteome length with these regions (%) are shown. ARs, amyloidogenic regions; Q, glutamine; N, asparagine; CBRs, compositionally biased regions; SARP, Sequence Analysis based on the Ranking of Probabilities.

To assess the abundance of potentially amyloidogenic proteins in plant proteomes, the proteins of 75 plant species available in the Uniprot Proteomes database (available at http://www.uniprot.org/proteomes/) were analyzed for the presence of amyloidogenic regions with two different bioinformatic approaches: Waltz, which predicts short amyloidogenic regions (ARs) based on a position-specific scoring matrix [37], and SARP, which searches for compositionally biased potentially amyloidogenic regions (CBRs) rich in particular residues [43]. For each proteome, we calculated the following: (i) fraction of potentially amyloidogenic proteins in the proteome; and (ii) the coverage of total proteome length with ARs and QN-rich CBRs (Figure 1, Table S1a).

Amyloidogenic regions (ARs) predicted by Waltz are abundant in the proteomes of plants. More than half of all proteins in each proteome contained at least one such region (Figure S1). Most ARs are very short at approximately 6–9 amino acids long, with a modal length of seven residues (Figure S2). Though such regions are amyloid-prone themselves [37], they may not contribute to amyloid-forming properties of the full-length proteins due to their short lengths. Therefore, to enhance the specificity of the predictions, we excluded from the Waltz analysis all ARs shorter than 10 amino acids. After this filtering, the median percentage of plant proteins that contained ARs predicted by Waltz was 25.41% (Table S1a). Potentially amyloidogenic compositionally biased regions (CBRs) predicted by SARP were significantly less abundant than ARs predicted by Waltz: approximately 1.38% of plant proteins contain QN-rich CBRs. The median length of CBRs predicted by SARP in plant proteomes was 203 residues for QN-rich CBRs (Table S1a). In contrast to potentially amyloidogenic proteins predicted by Waltz, most of the potentially amyloidogenic proteins predicted by SARP contained only one potentially amyloidogenic compositionally biased region. Notably, though amyloidogenic region predictions by Waltz and SARP were completely different, ARs predicted by Waltz were associated with CBRs rich in hydrophobic residues I, W, Y, F predicted by SARP (Figure S3). This result corresponds with the previous observation that amino acids with hydrophobic side chains have the highest amyloidogenic potential (i.e., propensity to form amyloid structure) [36].

The AR contents predicted by Waltz and SARP varied broadly in the proteomes of different plant species and may be significantly different even in closely related species (Figure 1, Table S1a). For example, *Gossypium arboreum* has many fewer proteins containing ARs predicted by Waltz (20.6%) compared to *Gossypium hirsutum* (29.5%) (Figure 1), which originated as a hybrid of *Gossypium arboreum* and *Gossypium raimondii* [52]. Species of *Oryza* spp. significantly differ from one another in the content of proteins with QN-rich CBRs (Figure 1). We excluded *Ipomeae nil* from analysis because its proteome, available at Uniprot (Table S2), contained only proteins encoded by the chloroplast or mitochondrial genomes. The only conifer species, *Picea glauca*, drastically differed from other species in AR and QN-rich CBR contents (Figure 1), but this could be associated with an incomplete proteome available at Uniprot (Table S2). Despite variability in the content of ARs and QN-rich CBRs in the proteomes of land plants, there is a common tendency of the proteomes of grasses to have a lower percentage of proteins with ARs predicted by Waltz and to be more abundant in QN-rich proteins (Figure 1). It should be noted that the proteomes of plants have similar contents of potentially amyloidogenic proteins compared with the *Escherichia coli*, *Saccharomyces cerevisiae* and *Homo sapiens* proteomes (Table S1b), in which experimentally verified amyloid proteins have been previously reported [22,53,54]. Moreover, since plants have very large proteomes, the total number of potentially amyloidogenic proteins in several species of plants is greater even than the corresponding number in the human proteome (Table S1a,b).

### 2.2. Molecular Functions of Potentially Amyloidogenic Proteins of Plants

**Figure 2:**
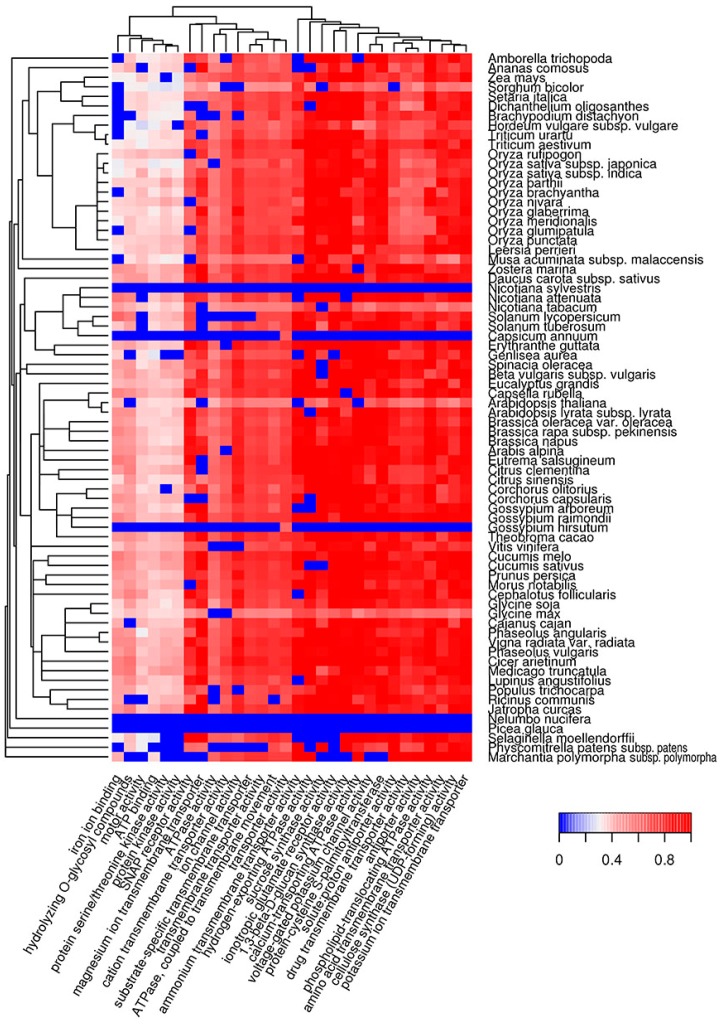
Heat map of GO molecular functions in which potentially amyloidogenic proteins predicted by Waltz are overrepresented. For such proteins, the top 30 GO terms from the molecular function ontology are shown. The color of the cells denotes the fraction of potentially amyloidogenic proteins predicted by Waltz among all proteins annotated with this term. All cells with *p*-values greater than 0.01 have values of 0 (**dark blue**). The dendrogram of plant species corresponds to their phylogenetic tree. GO, Gene Ontology.

**Figure 3:**
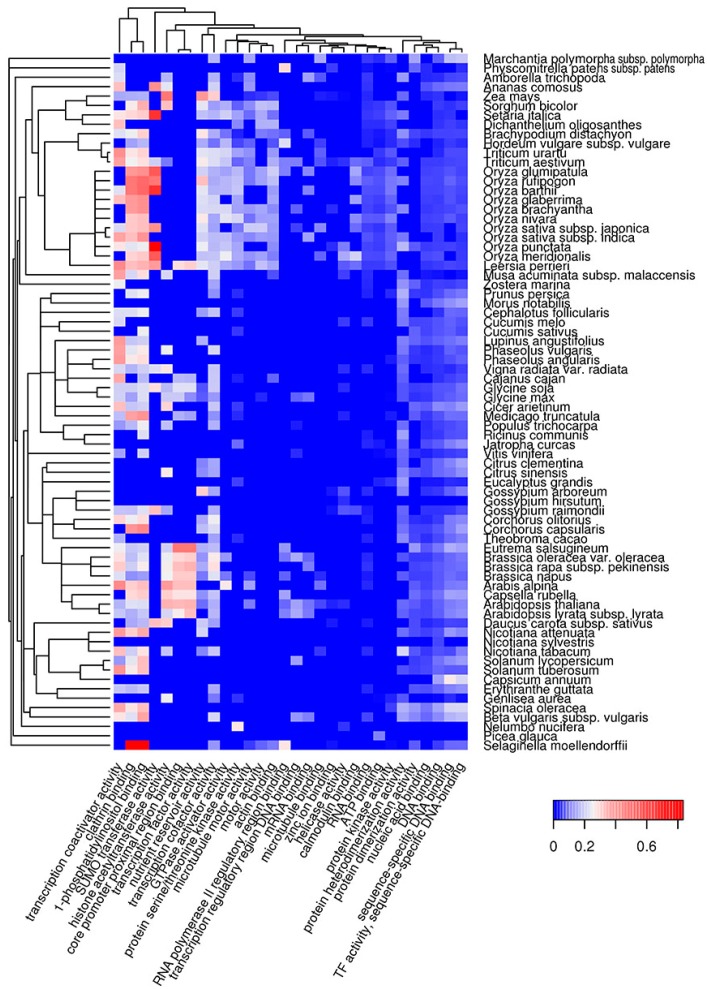
Heat map of GO molecular functions in which potentially amyloidogenic proteins containing QN-rich CBRs predicted by SARP are overrepresented. For such proteins, the top 30 GO terms from the molecular function ontology are shown. The color of the cells denotes the fraction of potentially amyloidogenic proteins predicted by SARP among all proteins annotated with this term. All cells with *p*-values greater than 0.01 have values of 0 (**dark blue**). The dendrogram of plant species corresponds to their phylogenetic tree.

Functional amyloids participate in diverse molecular functions in a wide spectrum of prokaryotic and eukaryotic species [13,54,55]. Functional amyloids may be active in the amyloid state [23,24,25,28] or act as protein or peptide storage reservoirs [26]. Thus, it was important to analyze the molecular functions of the predicted potentially amyloidogenic plant proteins to reveal functions that could be associated with amyloid formation. We searched for Gene Ontology (GO) terms related to molecular functions where potentially amyloidogenic proteins detected by Waltz and SARP are overrepresented. We found that GO terms enriched in proteins harboring ARs predicted by Waltz were drastically different from the terms associated with QN-rich proteins predicted by SARP. For instance, amyloidogenic regions predicted by Waltz were found mostly in transmembrane proteins with transporter activity as well as proteins with motor and kinase activities (Figure 2, Table S1c). Conversely, proteins harboring QN-rich CBRs were mostly associated with transcription, DNA- and RNA-binding activities, and protein oligomerization (Figure 3, Table S1d). Both ARs and QN-rich CBRs-containing proteins shared kinase activity as a function (Figure 2 and Figure 3). Several molecular functions were specific to particular systematic groups. For example, microtubule motor and actin-binding activities were characteristic of *Poaceae* QN-rich proteins (Figure 3). Notably, QN-rich proteins of approximately two-thirds of the analyzed species were associated with nutrient reservoir activity. Proteins with this function belong mostly to seed storage proteins that are known to be rich in Q and E in several species [56,57].

Since E-rich proteins are also potentially amyloidogenic, we analyzed GO molecular functions associated with plant proteins containing E-rich CBRs predicted by SARP (Figure S4, Table S1e). Several functions of E-rich proteins were found to be similar to those observed for QN-rich proteins including nucleic acid and clathrin binding. In contrast to QN-rich proteins, in which microtubule motor and actin binding activities were typical only for *Poaceae* proteins, E-rich proteins harboring these functions were characteristic of most plant species analyzed (Figure S4, Table S1e). Some functions, including translation-associated activities and unfolded protein binding, were specific to E-rich proteins (Figure S4). Finally, E-rich proteins with nutrient reservoir activity were abundant in fewer plant species compared to QN-rich proteins (Figure 3 and Figure S4). Thus, the molecular functions of potentially amyloidogenic proteins predicted by Waltz drastically differ from the functions of potentially amyloidogenic QN- and E-rich proteins that are partially similar.

### 2.3. Subcellular Localization of Potentially Amyloidogenic Proteins of Plants

We analyzed distribution of amyloidogenic proteins over different cellular components according to the Gene Ontology database (available at http://www.geneontology.org/). Potentially amyloidogenic proteins harboring ARs predicted by Waltz were found to be associated with different membranes, membrane organelles, myosin and V-type ATPase complexes (Figure S5, Table S1f). Potentially amyloidogenic proteins with QN-rich CBRs were associated with the RNA polymerase II transcription complex, nucleus, RNA-processing complexes, cytoskeleton and clathrin-coated vesicles (Figure S6, Table S1g). Interestingly, QN-rich proteins were abundant among proteins of P-bodies of only Asian species of rice, but not in the African species (Figure S6, Table S1g). Potentially amyloidogenic proteins with E-rich CBRs were associated with the translation machinery complex, cytoskeleton and chromosomes (Figure S7, Table S1h). Overall, the cellular components where different types of potentially amyloidogenic proteins predominate correspond to the molecular functions of these proteins. The general tendency is that potentially amyloidogenic proteins predicted by Waltz have membrane localization, while potentially amyloidogenic proteins with QN- and E-rich CBRs predicted by SARP are mainly cytoplasmic or intranuclear.

### 2.4. Biological Processes Implementing Potentially Amyloidogenic Proteins of Plants

**Figure 4:**
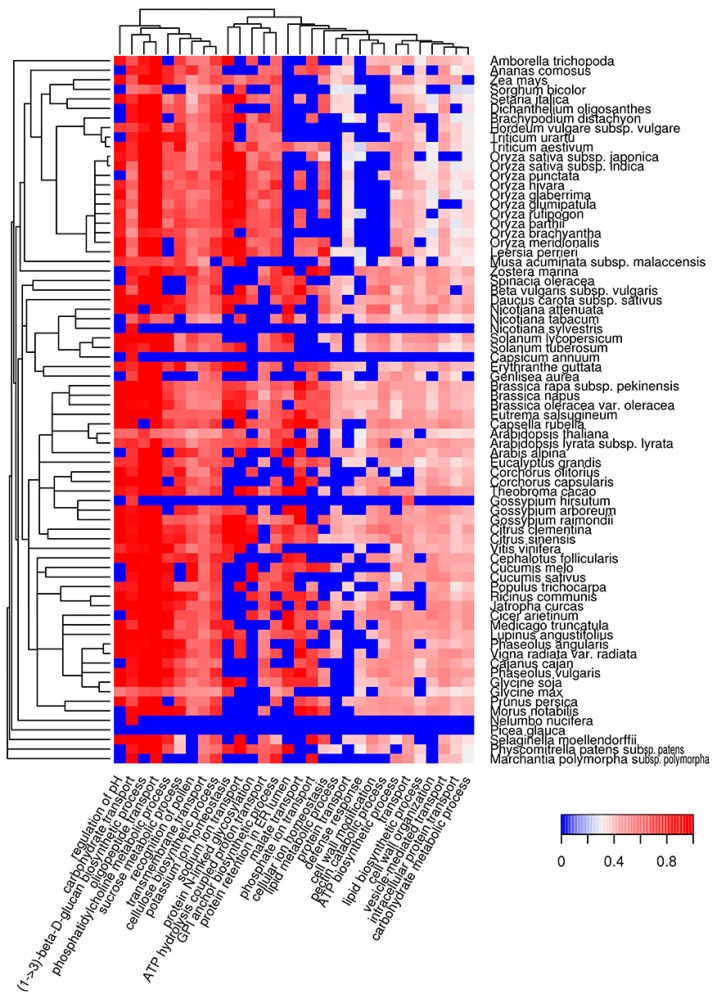
Heat map of GO biological processes in which potentially amyloidogenic proteins predicted by Waltz are overrepresented. For such proteins, the top 30 GO terms from the molecular function ontology are shown. The color of the cells denotes the fraction of potentially amyloidogenic proteins predicted by Waltz among all proteins annotated with this term. All cells with *p*-values greater than 0.01 have values of 0 (**dark blue**). The dendrogram of plant species corresponds to their phylogenetic tree.

We characterized the molecular functions and subcellular localization of potentially amyloidogenic proteins of different plant species. As a next step, we analyzed biological processes in which potentially amyloidogenic proteins participate. We found that proteins with ARs predicted by Waltz are overrepresented in biological processes associated with transmembrane transport, such as regulation of pH and ion (sodium, potassium, phosphate) and carbohydrate transport (Figure 4, Table S1i). Among these, there are several processes related to biosynthesis (cellulose and lipid biosynthesis, cell wall modifications) or associated with responses to outer factors (recognition of pollen and defense response). Interestingly, the defense response is a biological process in which Waltz-predicted potentially amyloidogenic proteins are abundant in the majority of plant species, with the exception of most grasses (Figure 4).

**Figure 5:**
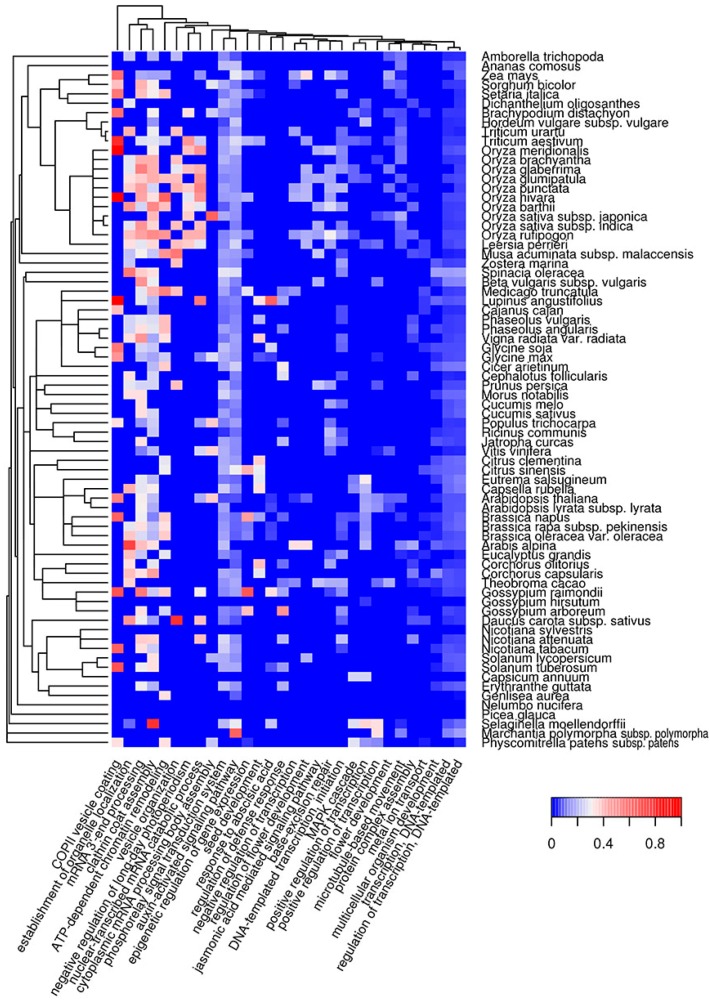
Heat map of GO biological processes in which QN-rich potentially amyloidogenic proteins predicted by SARP are overrepresented. For such proteins, the top 30 GO terms from the molecular function ontology are shown. The color of the cells denotes the fraction of QN-rich potentially amyloidogenic proteins predicted by SARP among all proteins annotated with this term. All cells with *p*-values greater than 0.01 have values of 0 (**dark blue**). The dendrogram of plant species corresponds to their phylogenetic tree.

The biological processes in which QN-rich potentially amyloidogenic proteins are abundant are mostly related to transcription, cytoskeleton organization and clathrin vesicle formation (Figure 5, Table S1j). Some are connected with the regulation of development, such as the negative regulation of long-day photoperiodism, seed and flower development, auxin, jasmonic and abscisic acid pathways (Figure 5, Table S1j). Overrepresentation of potentially amyloidogenic proteins in some of these processes can only occur in a few species. For example, the flower development process is only associated with QN-rich proteins in several very distant plant species: *Arabidopsis* spp., *Teobroma cacao*, *Vitis vinifera*, *Amborella trichopoda* and some grasses. Similar to QN-rich proteins, potentially amyloidogenic E-rich proteins are associated with the cytoskeleton and genome organization, as well as RNA processing (Figure S8, Table S1k). However, E-rich proteins are also overrepresented among the translation initiation and folding machinery components (Figure S8, Table S1k). Taken together, QN-rich proteins are similar to E-rich proteins for subcellular localizations, but each of the three groups of potentially amyloidogenic proteins (Waltz-predicted, QN-rich and E-rich) is involved in specific molecular functions and biological processes that only partially overlap.

### 2.5. Amyloidogenic Proteins in the Chloroplast and Mitochondrial Proteomes of Different Plant Species

**Figure 6:**
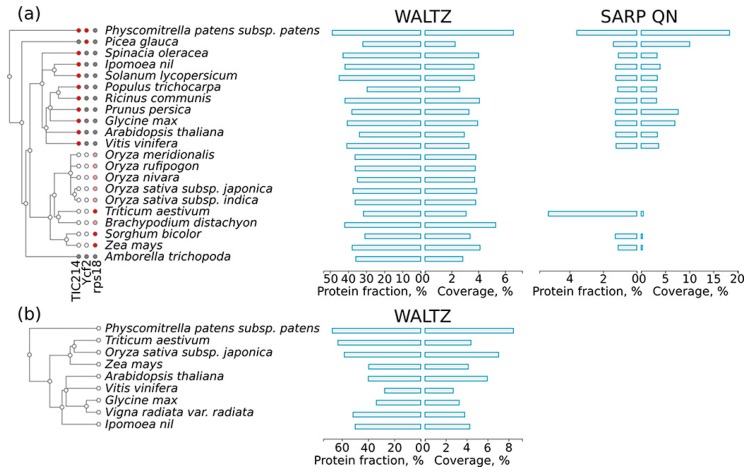
(**a**) Distribution of chloroplast sequences potentially capable of forming amyloids across land plant proteins. A taxonomic tree of plant species is shown according to the Uniprot Taxonomy. The results for amyloidogenic regions predicted by Waltz and QN-rich sequences found with SARP are shown. For each type of amyloidogenic region, the fraction of proteins harboring these regions and the coverage of the total proteome length with these regions are shown. For the TIC214, Ycf2 and rps18 proteins: (i) a red circle means that the protein is present in the proteome and has a QN-rich region; (ii) a gray circle denotes that the protein is encoded by the chloroplast genome but lacks a QN-rich region; (iii) a white circle denotes that there is no corresponding gene in the chloroplast genome; and (iv) a pink circle denotes that the rps18 protein has a small, manually verified QN-rich region. (**b**) Distribution of potentially amyloidogenic regions across higher plant proteins encoded by the mitochondrion genome. A taxonomic tree of plant species is shown according to the Uniprot Taxonomy. The results for Waltz-predicted regions are shown. For each type of amyloidogenic region, the fraction of proteins harboring these regions and the coverage of the total proteome length with these regions are shown. The results for QN-rich proteins predicted by SARP are not shown since such proteins are absent in the proteome of the mitochondrion.

Proteins encoded in the organellar genomes might be very different from proteins encoded in the nuclear genome. Therefore, we separately analyzed the distribution of potentially amyloidogenic proteins among the proteins encoded by the chloroplast and mitochondrial genomes. We found that proteins encoded in the organellar genomes have more regions predicted by Waltz in both the chloroplast and mitochondrion proteomes (Figure 6a,b) compared to the nuclear genome encoded proteins of the same species (Figure 1). At the same time, only three chloroplast proteins (Figure 6a) and no mitochondrial proteins contained QN-rich regions. These three proteins encoded in the chloroplast genome demonstrate interesting variability in the presence of QN-rich regions. The first is TIC214, the only component of the translocon at the chloroplast inner envelope [58]. It is present in most land plant species with the exception of grasses [59] (Figure 6a) and has a long QN-rich region in its C-terminus. The second chloroplast protein, Ycf2, has a QN-rich region only in Bryophyta (spreading earth moss, *Psycomitrella patens*) and Pinophyta (white spruce, *Picea glauca*) species, but not in the flowering plants. The third protein, an omnipresent ribosomal protein of the small subunit, rps18, has a short QN-rich region only in grasses. The QN-rich region of rps18 in many species of grasses was too short to be detected with SARP, but it was validated manually. Taken together, proteins encoded in the organellar genomes are enriched with potentially amyloidogenic proteins predicted by Waltz, while chloroplast QN-rich proteins show evolutionary conservation of their amyloidogenic regions.

### 2.6. Co-Occurrence of Potentially Amyloidogenic Regions with the Structural Features of Proteins

**Figure 7:**
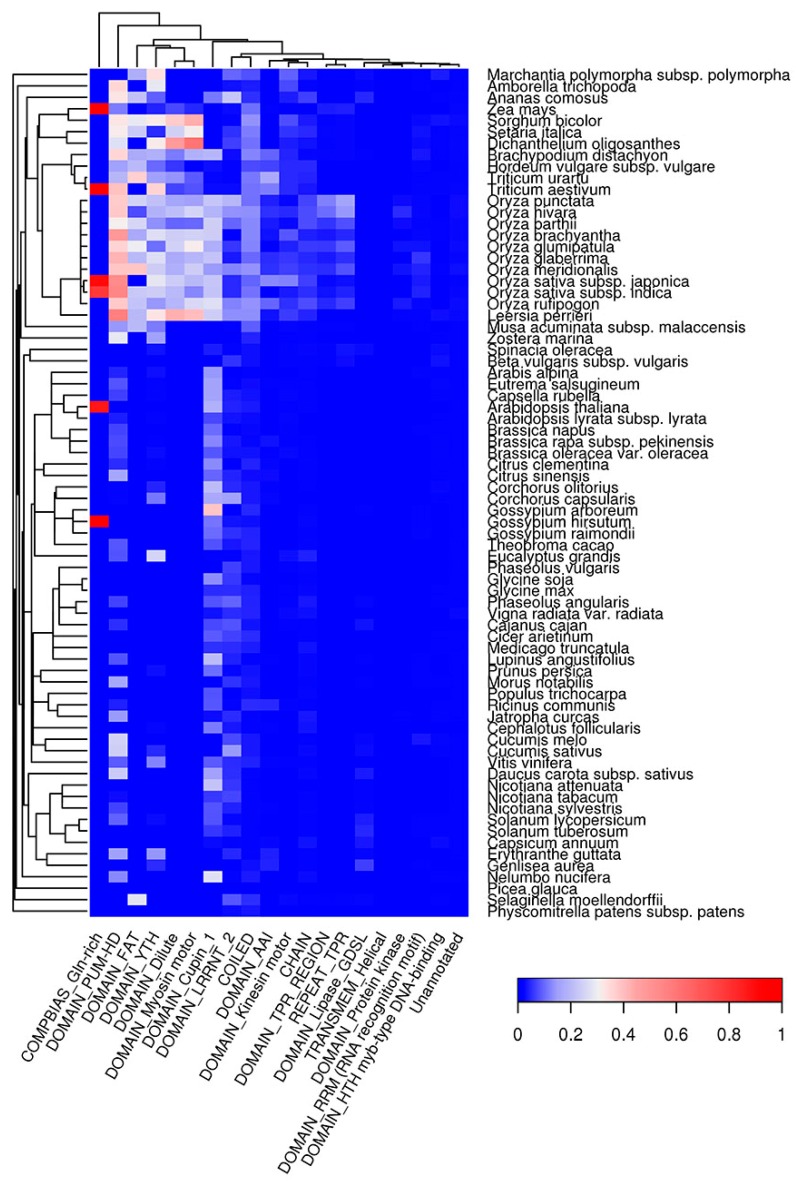
Top 20 protein features that are overrepresented in QN-rich regions predicted with SARP. The color of the cells denotes the fraction of proteins with amyloidogenic regions among all proteins with this feature. The dendrogram of plant species corresponds to their phylogenetic tree.

Potentially amyloidogenic regions have specific amino acid compositions and physical properties, and thus they might tend to be incorporated into certain structural features of proteins. We analyzed co-occurrence of QN-rich regions and regions predicted with Waltz with different types of protein domains. We found that QN-rich regions tend to co-occur with different DNA- (HTH Myb-type) and RNA-binding (YTH, RRM, PUM-HD), kinase (FAT), lipase (GDSL), and cytoskeleton-related domains (Dilute, Myosin, Kinesin) (Figure 7). QN-rich regions were also found to be associated with the LRRNT domain, which is mostly responsible for protein-protein interactions [60]. Importantly, in many plant species, the QN-rich regions overlap with the conserved barrel domain, Cupin1, of the 11S and 7S plant seed storage proteins. For deeper analysis of the association between seed storage protein domains and QN-rich regions, we used PFAM database (see Section 4.7) [61]. We found that 302 storage proteins with Cupin1 were Q/N-rich in 54 of 75 plant species analyzed (Table 1). Q/N-rich storage proteins containing other domains were less abundant. For example, we detected 119 Q/N-rich proteins with Zein domain in three plant species; 121 with Gliadin domain in 15 species; 13 with Vicilin domain in nine species; and seven with high molecular weight Glutenin in two plant species analyzed (Table 1). Taken together, our data show that different seed storage proteins in various plant species are associated with the presence of potentially amyloidogenic Q/N-rich regions.

**Figure 8:**
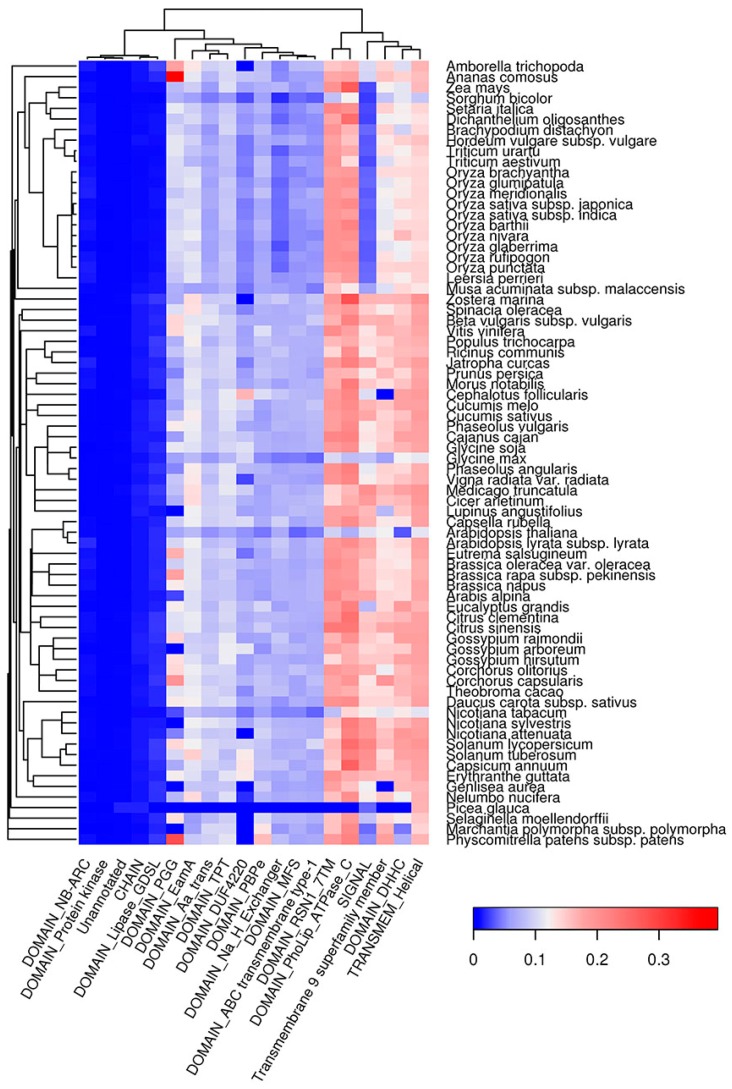
Top 20 protein features that are overrepresented in Waltz-predicted amyloidogenic regions. The color of the cells denotes the fraction of proteins with amyloidogenic regions among all proteins with this feature. The dendrogram of plant species corresponds to their phylogenetic tree.

Similar to QN-rich regions, E-rich regions of plant proteins were mainly enriched with DNA-binding (HMG, SMC) and cytoskeleton-associated (NAB, Kinesin) domains (Figure S9). Additionally, E-rich regions were associated with Helicase and Cactin domains as well as with GTD and FF domains, which are likely responsible for protein-protein interactions (Figure S9). In contrast to QN- and E-rich regions, amyloidogenic regions predicted with Waltz tend to be inside transmembrane domains (EamA, TPT, PBPe, MFS, ABC transmembrane Type-1, etc.) in all plant species analyzed except for *P. glauca* (Figure 8), which is likely because of incomplete proteome annotation for this species. Signal peptides were strongly associated with ARs predicted by Waltz in all species except grasses (Figure 8). Notably, both QN-rich regions and ARs predicted by Waltz are associated with protein kinase domains (Figure 7 and Figure 8). Thus, amyloidogenic regions occupy specific protein domains (Figure 7, Figure 8 and Figure S9), which might reflect the involvement of ARs in the functioning of these domains.

## 3. Discussion

The bioinformatic analysis performed in this study revealed that potentially amyloidogenic proteins are abundant in the proteomes of land plants (Figure 1). These proteins exhibit various molecular functions, cellular localizations and biological processes (Figure 2, Figure 3, Figure 4 and Figure 5). Two algorithms used in our study, Waltz and SARP, revealed different groups of potentially amyloidogenic plant proteins based on their primary structure. Some of these proteins are related to amyloid-forming proteins in other groups of organisms identified in vivo or plant proteins whose amyloid properties were partially characterized in vitro and in heterologous systems.

Most groups of plant proteins predicted by Waltz are transmembrane proteins acting as transporters of different compounds. Such proteins can potentially have amyloid properties. For example, porins OmpA and OmpC of the bacteria *Escherichia coli* were shown to have amyloid properties [62,63]. Thus, we cannot exclude that several membrane proteins of plants could also adopt amyloid structures. The second group of amyloidogenic proteins predicted by Waltz to be abundant in most of the species analyzed were defense proteins. These proteins represent a large and heterogeneous group, many representatives of which are hydrophobic [64]. Interestingly, several plant defense proteins and peptides were shown to have amyloid-like properties in vitro [49,50,65]. Amyloid formation by such plant proteins could stabilize them and enhance their survival during interactions with pathogens, since amyloids are extremely stable [66].

Amyloidogenic proteins of plants predicted with SARP were mainly localized in the nucleus and cytoplasm. In the case of QN-rich plant proteins, DNA- and RNA-binding activities including transcriptional regulation are the most common. There are numerous examples of Q and/or N-rich transcriptional factors among human and yeast amyloid-forming proteins [38]. Moreover, Luminidependens, a QN-rich transcriptional regulator of flowering in *Arabidopsis thaliana*, was recently shown to have amyloid- and prion-like properties in a heterologous yeast system [46]. We also found that QN-rich proteins are overrepresented among floral regulators, but only in several species including *A. thaliana* (Figure 5). Overall, according to bioinformatic data, DNA- and RNA-binding QN-rich proteins of plants represent a promising group to search for novel amyloid-forming proteins. The second group of potentially amyloidogenic proteins predicted by SARP was E-rich, which were similar to QN-rich in function and localization, but additionally included translation- and folding-related proteins (Figure S8) that could be involved in amyloid formation.

One of the most important findings of this study was the overrepresentation in different plant species of potentially amyloidogenic proteins among proteins acting as nutrient reservoirs (Figure 3 and Figure S4), including seed storage proteins, which constitute an important part of the human diet. Moreover, the evolutionarily conserved Cupin1 as well as Zein, Gliadin, Vicilin and high molecular weight Glutenin domains of seed storage proteins tend to have potentially amyloidogenic QN-rich regions (Figure 7, Table 1). Previously, proteolytic peptides of seed storage proteins of leguminous plants were shown to form fibrils with several properties of amyloids in vitro [67,68,69]. Based on these observations, we hypothesized that storage proteins might adopt amyloid states in seeds to accumulate and stabilize their molecules during dehydration that naturally occurs as a result of seed maturation [51]. The data obtained in this study strongly support our hypothesis. We may expect that the process of accumulation of storage proteins in the seeds could be similar to the accumulation of human hormones in the amyloid state [26] or dehydration-dependent amyloid formation by the proteins of egg envelop of “annual killfish” *Austrofundulus limnaeus* [51,70].

We found that QN-rich proteins were absent in the mitochondria and that few chloroplast proteins contained QN-rich regions (Figure 6). One such protein is TIC214, which harbors a QN-rich region in its C-terminus in all investigated plant species (see Section 2.5). It should be noted that TIC214 is the only translocon component on the inner envelope of chloroplasts that is encoded in the chloroplast genome [59]. Though it is omnipresent in most species of plants (except grasses), the C-terminal region is highly variable. The only common feature of the C-terminal region of TIC214 in different species is the presence of charged motifs [59]. Possibly, an increased QN content might be important for interspersing these motifs. Another chloroplast protein, Ycf2, contains a QN-rich region, but not in the flowering plants (Figure 6). The changes in Ycf2 composition coincide with its gene duplication in the flowering plants lineage [71]. The *Poaceae* species have lost the Ycf1/TIC214 protein, but they have a small QN-rich region in the C-terminal region of the rps18 protein (Figure 6). These examples suggest that the composition of QN-rich regions might correspond with the evolution of species, even when the sequence of such regions is highly variable. Additionally, such a conservation of amino acid composition suggests that CBRs may be functionally important.

Undoubtedly, the presence of bioinformatically predicted amyloidogenic regions does not indicate that the corresponding full-length proteins have amyloid properties in vivo. Nevertheless, resistance of proteins to treatment with ionic detergents, which is one of the key properties of amyloids, correlates with the presence of ARs predicted by WALTZ and CBRs predicted by SARP [72], and the most of experimentally analyzed amyloidogenic plant proteins (LD, FPA, FCA, TGZ, monellin, pro-hevein) [51] bear such regions. Thus, predictions of potentially amyloidogenic proteins with these algorithms are useful not only to analyze molecular functions, subcellular functions, and domain structure of such proteins but also to reveal candidates in plant proteomes for experimental analysis of their amyloid-forming properties. Identification of novel amyloid proteins is laborious and time-consuming, but bioinformatic predictions in combination with recently developed proteomic approaches [72,73,74,75] are useful in this regard. In addition, future development of novel, more efficient bioinformatic algorithms based on the machine learning, which is actively using now for protein analysis [76,77], could also contribute to the progress in the proteomics of amyloids.

Overall, in this study, we have investigated the diversity of amyloidogenic proteins in plant species, analyzed their functions and localization, and, based on the obtained bioinformatic data, suggested possible roles of amyloid formation in different biological processes including defense from pathogens and storage of proteins in seeds.

## 4. Materials and Methods

### 4.1. Datasets

All protein sequences of 75 plant species were downloaded with their annotations from the Uniprot Proteomes database (available at http://www.uniprot.org/proteomes/). We used the sequences listed in the reference proteomes for these species in June of 2017. To fetch the data, we used the Proteins REST API (available at http://www.ebi.ac.uk/proteins/api/doc) [78]. Phylogenetic trees of plant species were obtained according to the Uniprot Taxonomy (available at http://www.uniprot.org/taxonomy/). IDs of the proteomes and taxonomies used are listed in Table S2.

### 4.2. Prediction of Amyloidogenic Regions

Prediction of amyloidogenic regions was performed using the Waltz algorithm [37], with parameters set as follows: threshold–best overall selectivity and pH 7.0. Protein sequences that did not match the Waltz requirements (sequence should not contain uncanonical amino acid letters and should not be longer ten thousand residues) were excluded. Proteins harboring at least one region predicted with Waltz longer than 9 amino acids were marked as potentially amyloidogenic proteins. Coverages of Waltz-predicted regions were calculated as follows: total length of all regions predicted by WALTZ divided by sum of lengths of all proteins in the corresponding proteome. A comparison of different species by the portion of potentially amyloidogenic proteins in the proteomes was performed with Fisher’s exact test [79] with a Benjamini and Hochberg *p*-value adjustment [80].

### 4.3. Prediction of Compositionally Biased Regions

Prediction of compositionally biased regions (CBRs) in proteins for E, Q and N amino acids was performed with the SARP algorithm [43]. The threshold of probability was set to 10^−8^. Calculations of coverage of CBRs and comparisons of different species by their proportion of compositionally biased regions in proteomes were performed as for ARs (see Section 4.1). The proteins were considered potentially amyloidogenic if they harbor at least one CBR rich in E or Q and N.

### 4.4. GO Term Enrichment Test

GO term enrichment tests were performed with the topGO R package [81]. Only terms with *p*-values less than 0.01 and at least five proteins in the list of interest were selected. All proteins in the proteome for each species were used as the protein universe, and only proteins with predicted amyloidogenic regions or compositionally biased regions were included in the list of proteins of interest. The heatmap.2 function from the gplots package was used to draw heat maps with default clustering functions.

### 4.5. Identification of Potentially Amyloidogenic Proteins in the Proteomes of Organelles

Data on whether proteins were encoded by mitochondrion or chloroplast genomes were obtained from the proteome annotation in the Uniport database. For each set of proteins, amyloidogenic regions were predicted with Waltz (see Section 4.2), and QN-rich CBRs were found with SARP (see Section 4.3). Statistics for the ARs and CBRs were calculated for each set separately, as described in Section 4.2 and Section 4.3.

### 4.6. Analysis of the Association between Amyloidogenic Regions and Different Protein Features

Feature annotation was obtained from the Uniprot database. All sequence regions that were not assigned to any feature were marked as unannotated. For each type of feature, the sum of the length of overlaps of all amyloidogenic regions, and amyloidogenic CBRs rich in QN or E with these features were calculated and divided by the total length of features of that type. The distribution of ARs predicted by Waltz over different CBRs was calculated the same way (summing the lengths of all ARs overlapping with CBRs of a given type and dividing by the total length of all CBRs of this type). The heatmap.2 function from the gplots package was used to draw heat maps with default clustering functions.

### 4.7. Analysis of the Abundance of the PFAM Domains among Proteins Containing CBRs

We used PFAM annotation for proteins from Uniprot database (available at http://www.uniprot.org/). The descriptions for PFAM families were fetched from PFAM database [61] (available at http://pfam.xfam.org/). To calculate the abundance of the PFAM domains among proteins with nutrient reservoir activity, we obtained the list of PFAM accessions associated with the proteins with GO:0045735 and calculate the number of proteins from this subset for each PFAM accession. The abundance of the PFAM domains among QN-rich proteins was calculated in the same way, but only proteins with GO:0045735 containing QN-rich regions predicted by SARP were selected. For each PFAM accession, we calculated the number of species in which proteomes proteins with corresponding PFAM domains from given subsets were present.

## Figures and Tables

**Table 1:** Distribution of the potentially amyloidogenic QN-rich storage proteins across the plant proteomes.

PFAM id	Domain Family Description	Number of PFAM Proteins *	Number of Species with PFAM Proteins	Number of QN-Rich Proteins	Number of Species with QN-Rich PFAM Proteins	Percentage of QN-Rich PFAM Proteins	Percentage of Species with QN-Rich PFAM Proteins
PF00190	Cupin	3973	70	302	54	7.60	77.14
PF01559	Zein seed storage protein	161	3	119	3	73.91	100.00
PF13016	Cys-rich Gliadin N-terminal	133	16	121	15	90.98	93.75
PF00234	Protease inhibitor/seed storage/LTP family	113	28	52	15	46.02	53.57
PF01535	PPR repeat	18	8	13	5	72.22	62.50
PF13041	PPR repeat family	17	8	13	5	76.47	62.50
PF04702	Vicilin N terminal region	17	13	13	9	76.47	69.23
PF12854	PPR repeat	16	7	13	5	81.25	71.43
PF03157	High molecular weight glutenin subunit	9	3	7	2	77.78	66.67
PF13639	Ring finger domain	7	6	7	6	100.00	100.00
PF03330	Lytic transglycolase	2	1	1	1	50.00	100.00
PF01357	Pollen allergen	2	1	1	1	50.00	100.00
PF13446	A repeated domain in UCH-protein	1	1	1	1	100.00	100.00
PF03145	Seven in absentia protein family	1	1	1	1	100.00	100.00
Total		4487	70	601	59	13.39	84.29

* PFAM protein: the storage protein (GO:0045735) containing corresponding domain belonging to the PFAM family indicated in the column “Domain family description”. Q: glutamine; N: asparagine.

## Supplementary Materials

Supplementary materials can be found at www.mdpi.com/1422-0067/18/10/2155/s1.

## Abbreviations

AR	Amyloidogenic region
SARP	Sequence Analysis Based on the Ranking of Probabilities
CBR	compositionally biased region

## References

[1] Sipe J.D., Cohen A.S. (2000). Review: History of the amyloid fibril. J. Struct. Biol..

[2] Eanes E.D., Glenner G.G. (1968). X-ray diffraction studies on amyloid filaments. J. Histochem. Cytochem..

[3] Tycko R., Wickner R.B. (2013). Molecular structures of amyloid and prion fibrils: Consensus versus controversy. Acc. Chem. Res..

[4] Selkoe D.J., Ihara Y., Salazar F.J. (1982). Alzheimer’s disease: Insolubility of partially purified paired helical filaments in sodium dodecyl sulfate and urea. Science.

[5] Hazeki N., Tukamoto T., Goto J., Kanazawa I. (2000). Formic acid dissolves aggregates of an N-terminal huntingtin fragment containing an expanded polyglutamine tract: Applying to quantification of protein components of the aggregates. Biochem. Biophys. Res. Commun..

[6] Bolton D.C., McKinley M.P., Prusiner S.B. (1982). Identification of a protein that purifies with the scrapie prion. Science.

[7] Kyle R.A. (2001). Amyloidosis: A convoluted story. Br. J. Haematol..

[8] Virchow R. (1854). Ueber eine im Gehirn und Ruckenmark des Menschen aufgefunde Substanz mit der chemishen Reaction der Cellulose. Virchows Arch. Path. Anat. Physiol..

[9] Friedreich N., Kekule F.A. (1859). Zur Amyloidfrage. Virchows Arch. Path. Anat. Physiol..

[10] Buxbaum J.N., Linke R.P. (2012). A molecular history of the amyloidoses. J. Mol. Biol..

[11] Chiti F., Dobson C.M. (2017). Protein Misfolding, Amyloid Formation, and Human Disease: A Summary of Progress Over the Last Decade. Annu. Rev. Biochem..

[12] Sipe J.D., Benson M.D., Buxbaum J.N., Ikeda S., Merlini G., Saraiva M.J., Westermark P. (2014). Nomenclature 2014: Amyloid fibril proteins and clinical classification of the amyloidosis. Amyloid.

[13] Pham C.L.L., Kwan A.H., Sunde M. (2014). Functional amyloid: Widespread in nature, diverse in purpose. Essays Biochem..

[14] Kelly J.W., Balch W.E. (2003). Amyloid as a natural product. J. Cell Biol..

[15] Chapman M.R., Robinson L.S., Pinkner J.S., Roth R., Heuser J., Hammar M., Normark S., Hultgren S.J. (2002). Role of *Escherichia coli* curli operons in directing amyloid fiber formation. Science.

[16] Bieler S., Estrada L., Lagos R., Baeza M., Castilla J., Soto C. (2005). Amyloid formation modulates the biological activity of a bacterial protein. J. Biol. Chem..

[17] Claessen D., Rink R., De Jong W., Siebring J., De Vreugd P., Boersma F.G.H., Dijkhuizen L., Wosten H.A.B. (2003). A novel class of secreted hydrophobic proteins is involved in aerial hyphae formation in *Streptomyces coelicolor* by forming amyloid-like fibrils. Genes Dev..

[18] Chimileski S., Franklin M.J., Papke R.T. (2014). Biofilms formed by the archaeon *Haloferax volcanii* exhibit cellular differentiation and social motility, and facilitate horizontal gene transfer. BMC Biol..

[19] Dueholm M.S., Larsen P., Finster K., Stenvang M.R., Christiansen G., Vad B.S., Boggild A., Otzen D.E., Nielsen P.H. (2015). The tubular sheaths encasing methanosaeta thermophila filaments are functional amyloids. J. Biol. Chem..

[20] Coustou V., Deleu C., Saupe S., Begueret J. (1997). The protein product of the het-s heterokaryon incompatibility gene of the fungus Podospora anserina behaves as a prion analog. Proc. Natl. Acad. Sci. USA.

[21] Holmes D.L., Lancaster A.K., Lindquist S., Halfmann R. (2013). Heritable remodeling of yeast multicellularity by an environmentally responsive prion. Cell.

[22] Gebbink M.F.B.G., Claessen D., Bouma B., Dijkhuizen L., Wösten H.A.B. (2005). Amyloids—A functional coat for microorganisms. Nat. Rev. Microbiol..

[23] Si K., Giustetto M., Etkin A., Hsu R., Janisiewicz A.M., Miniaci M.C., Kim J.H., Zhu H., Kandel E.R. (2003). A neuronal isoform of cpeb regulates local protein synthesis and stabilizes synapse-specific long-term facilitation in aplysia. Cell.

[24] Majumdar A., Cesario W.C., White-Grindley E., Jiang H., Ren F., Khan M.R., Li L., Choi E.M.L., Kannan K., Guo F. (2012). Critical role of amyloid-like oligomers of Drosophila Orb2 in the persistence of memory. Cell.

[25] Fowler D.M., Koulov A.V., Alory-Jost C., Marks M.S., Balch W.E., Kelly J.W. (2006). Functional amyloid formation within mammalian tissue. PLoS Biol..

[26] Maji S.K., Perrin M.H., Sawaya M.R., Jessberger S., Vadodaria K., Rissman R.A., Singru P.S., Nilsson K.P., Simon R., Schubert D. (2009). Functional amyloids as natural storage of peptide hormones in pituitary secretory granules. Science.

[27] Carneiro K.M.M., Zhai H., Zhu L., Horst J.A., Sitlin M., Nguyen M., Wagner M., Simpliciano C., Milder M., Chen C.-L. (2016). Amyloid-like ribbons of amelogenins in enamel mineralization. Sci. Rep..

[28] Cai X., Chen J., Xu H., Liu S., Jiang Q.X., Halfmann R., Chen Z.J. (2014). Prion-like polymerization underlies signal transduction in antiviral immune defense and inflammasome activation. Cell.

[29] Teng P.K., Eisenberg D. (2009). Short protein segments can drive a non-fibrillizing protein into the amyloid state. Protein Eng. Des. Sel..

[30] Von Bergen M., Friedhoff P., Biernat J., Heberle J., Mandelkow E.M., Mandelkow E. (2000). Assembly of tau protein into Alzheimer paired helical filaments depends on a local sequence motif ((306)VQIVYK(311)) forming β structure. Proc. Natl. Acad. Sci. USA.

[31] López de la Paz M., Serrano L. (2004). Sequence determinants of amyloid fibril formation. Proc. Natl. Acad. Sci. USA.

[32] Esteras-Chopo A., Serrano L., López de la Paz M. (2005). The amyloid stretch hypothesis: Recruiting proteins toward the dark side. Proc. Natl. Acad. Sci. USA.

[33] Kadnar M.L., Articov G., Derkatch I.L. (2010). Distinct type of transmission barrier revealed by study of multiple prion determinants of Rnq1. PLoS Genet..

[34] Das S., Pal U., Das S., Bagga K., Roy A., Mrigwani A., Maiti N.C. (2014). Sequence complexity of amyloidogenic regions in intrinsically disordered human proteins. PLoS ONE.

[35] Das A.K., Pandit R., Maiti S. (2015). Effect of amyloids on the vesicular machinery: Implications for somatic neurotransmission. Philos. Trans. R. Soc. Lond. B Biol. Sci..

[36] Ahmed A.B., Kajava A.V. (2013). Breaking the amyloidogenicity code: Methods to predict amyloids from amino acid sequence. FEBS Lett..

[37] Maurer-Stroh S., Debulpaep M., Kuemmerer N., Lopez de la Paz M., Martins I.C., Reumers J., Morris K.L., Copland A., Serpell L., Serrano L. (2010). Exploring the sequence determinants of amyloid structure using position-specific scoring matrices. Nat. Methods.

[38] Nizhnikov A.A., Antonets K.S., Bondarev S.A., Inge-Vechtomov S.G., Derkatch I.L. (2016). Prions, amyloids, and RNA: Pieces of a puzzle. Prion.

[39] Scherzinger E., Lurz R., Turmaine M., Mangiarini L., Hollenbach B., Hasenbank R., Bates G.P., Davies S.W., Lehrach H., Wanker E.E. (1997). Huntingtin-encoded polyglutamine expansions form amyloid-like protein aggregates in vitro and in vivo. Cell.

[40] Michelitsch M.D., Weissman J.S. (2000). A census of glutamine/asparagine-rich regions: Implications for their conserved function and the prediction of novel prions. Proc. Natl. Acad. Sci. USA.

[41] Colaco M., Park J., Blanch H. (2008). The kinetics of aggregation of poly-glutamic acid based polypeptides. Biophys. Chem..

[42] Harrison P.M., Gerstein M. (2003). A method to assess compositional bias in biological sequences and its application to prion-like glutamine/asparagine-rich domains in eukaryotic proteomes. Genome Biol..

[43] Antonets K.S., Nizhnikov A.A. (2013). SARP: A novel algorithm to assess compositional biases in protein sequences. Evol. Bioinform. Online.

[44] Beerten J., Van Durme J., Gallardo R., Capriotti E., Serpell L., Rousseau F., Schymkowitz J. (2014). WALTZ-DB: A benchmark database of amyloidogenic hexapeptides. Bioinformatics.

[45] Alberti S., Halfmann R., King O., Kapila A., Lindquist S. (2009). A systematic survey identifies prions and illuminates sequence features of prionogenic proteins. Cell.

[46] Chakrabortee S., Kayatekin C., Newby G.A., Mendillo M.L., Lancaster A., Lindquist S. (2016). Luminidependens (LD) is an Arabidopsis protein with prion behavior. Proc. Natl. Acad. Sci. USA.

[47] Yang W., Willemse J., Sawyer E.B., Lou F., Gong W., Zhang H., Gras S.L., Claessen D., Perrett S. (2017). The propensity of the bacterial rodlin protein RdlB to form amyloid fibrils determines its function in Streptomyces coelicolor. Sci. Rep..

[48] Macindoe I., Kwan A.H., Ren Q., Morris V.K., Yang W., Mackay J.P., Sunde M. (2012). Self-assembly of functional, amphipathic amyloid monolayers by the fungal hydrophobin EAS. Proc. Natl. Acad. Sci. USA.

[49] Gour S., Kaushik V., Kumar V., Bhat P., Yadav S.C., Yadav J.K. (2016). Antimicrobial peptide (Cn-AMP2) from liquid endosperm of Cocos nucifera forms amyloid-like fibrillar structure. J. Pept. Sci..

[50] Berthelot K., Lecomte S., Coulary-Salin B., Bentaleb A., Peruch F. (2016). Hevea brasiliensis prohevein possesses a conserved C-terminal domain with amyloid-like properties in vitro. Biochim. Biophys. Acta.

[51] Antonets K.S., Nizhnikov A.A. (2017). Amyloids and prions in plants: Facts and perspectives. Prion.

[52] Wendel J.F. (1989). New World tetraploid cottons contain Old World cytoplasm. Proc. Natl. Acad. Sci. USA.

[53] Wickner R.B., Shewmaker F.P., Bateman D.A., Edskes H.K., Gorkovskiy A., Dayani Y., Bezsonov E.E. (2015). Yeast prions: Structure, biology, and prion-handling systems. Microbiol. Mol. Biol. Rev..

[54] Nizhnikov A.A., Antonets K.S., Inge-Vechtomov S.G. (2015). Amyloids: From pathogenesis to function. Biochemistry.

[55] Fowler D.M., Koulov A.V., Balch W.E., Kelly J.W. (2007). Functional amyloid—From bacteria to humans. Trends Biochem. Sci..

[56] Balakireva A.V., Zamyatnin A.A. (2016). Properties of gluten intolerance: Gluten structure, evolution, pathogenicity and detoxification capabilities. Nutrients.

[57] Jackson P., Boulter D., Thurman D.A. (1969). A comparison of some properties of vicilin and legumin isolated from seeds of *Pisum sativum*, *Vicia faba* and *Cicer arietinum*. New Phytol..

[58] Kikuchi S., Bédard J., Hirano M., Hirabayashi Y., Oishi M., Imai M., Takase M., Ide T., Nakai M. (2013). Uncovering the protein translocon at the chloroplast inner envelope membrane. Science.

[59] De Vries J., Sousa F.L., Bölter B., Soll J., Gould S.B. (2015). YCF1: A Green TIC?. Plant Cell.

[60] Kobe B., Kajava A.V. (2001). The leucine-rich repeat as a protein recognition motif. Curr. Opin. Struct. Biol..

[61] Finn R.D., Coggill P., Eberhardt R.Y., Eddy S.R., Mistry J., Mitchell A.L., Potter S.C., Punta M., Qureshi M., Sangrador-Vegas A. (2016). The Pfam protein families database: Towards a more sustainable future. Nucleic Acids Res..

[62] Danoff E.J., Fleming K.G. (2015). Aqueous, Unfolded OmpA forms amyloid-like fibrils upon self-association. PLoS ONE.

[63] Joseph Sahaya Rajan J., Chinnappan Santiago T., Singaravel R., Ignacimuthu S. (2016). Outer membrane protein C (OmpC) of *Escherichia coli* induces neurodegeneration in mice by acting as an amyloid. Biotechnol. Lett..

[64] Nawrot R., Barylski J., Nowicki G., Broniarczyk J., Buchwald W., Goździcka-Józefiak A. (2014). Plant antimicrobial peptides. Folia Microbiol..

[65] Garvey M., Meehan S., Gras S.L., Schirra H.J., Craik D.J., van der Weerden N.L., Anderson M.A., Gerrard J.A., Carver J.A. (2013). A radish seed antifungal peptide with a high amyloid fibril-forming propensity. Biochim. Biophys. Acta Proteins Proteom..

[66] Wiggins R.C. (2009). Prion Stability and infectivity in the environment. Neurochem. Res..

[67] Munialo C.D., Martin A.H., van der Linden E., de Jongh H.H.J. (2014). Fibril formation from pea protein and subsequent gel formation. J. Agric. Food Chem..

[68] Tang C.H., Wang C.S. (2010). Formation and characterization of amyloid-like fibrils from soy β-conglycinin and glycinin. J. Agric. Food Chem..

[69] Ridgley D.M., Ebanks K.C., Barone J.R. (2011). Peptide mixtures can self-assemble into large amyloid fibers of varying size and morphology. Biomacromolecules.

[70] Podrabsky J.E., Carpenter J.F., Hand S.C. (2001). Survival of water stress in annual fish embryos: Dehydration avoidance and egg envelope amyloid fibers. Am. J. Physiol. Regul. Integr. Comp. Physiol..

[71] Wolf P.G., Der J.P., Duffy A.M., Davidson J.B., Grusz A.L., Pryer K.M. (2011). The evolution of chloroplast genes and genomes in ferns. Plant Mol. Biol..

[72] Antonets K.S., Volkov K.V., Maltseva A.L., Arshakian L.M., Galkin A.P., Nizhnikov A.A. (2016). Proteomic analysis of *Escherichia coli* protein fractions resistant to solubilization by ionic detergents. Biochemistry.

[73] Nizhnikov A.A., Alexandrov A.I., Ryzhova T.A., Mitkevich O.V., Dergalev A.A., Ter-Avanesyan M.D., Galkin A.P. (2014). Proteomic screening for amyloid proteins. PLoS ONE.

[74] Nizhnikov A.A., Ryzhova T.A., Volkov K.V., Zadorsky S.P., Sopova J.V., Inge-Vechtomov S.G., Galkin A.P. (2016). Interaction of Prions Causes Heritable Traits in *Saccharomyces cerevisiae*. PLOS Genet..

[75] Kryndushkin D., Pripuzova N., Burnett B.G., Shewmaker F. (2013). Non-targeted identification of prions and amyloid-forming proteins from yeast and mammalian cells. J. Biol. Chem..

[76] Wan S., Duan Y., Zou Q. (2017). HPSLPred: An Ensemble Multi-Label Classifier for Human Protein Subcellular Location Prediction with Imbalanced Source. Proteomics.

[77] Liao Z., Wang X., Zeng Y., Zou Q. (2016). Identification of DEP domain-containing proteins by a machine learning method and experimental analysis of their expression in human HCC tissues. Sci. Rep..

[78] Nightingale A., Antunes R., Alpi E., Bursteinas B., Gonzales L., Liu W., Luo J., Qi G., Turner E., Martin M. (2017). The Proteins API: Accessing key integrated protein and genome information. Nucleic Acids Res..

[79] Fisher R.A. (1932). The logic of inductive inference. J. R. Stat. Soc..

[80] Benjamini Y., Hochberg Y. (1995). Controlling the False Discovery Rate: A Practical and Powerful Approach to Multiple Testing. J. R. Stat. Soc. Ser. B.

[81] Alexa A., Rahnenfuhrer J. (2016). topGO: Enrichment Analysis for Gene Ontology. R package version 2.28.0. Bioconductor.

