# Supplementary figures and images for: Predicting Amyloidogenic Proteins in the Proteomes of Plants
Source: Int J Mol Sci. 2017 Oct 16;18(10):2155. doi: 10.3390/ijms18102155 (PMC5666836; doi:10.3390/ijms18102155)

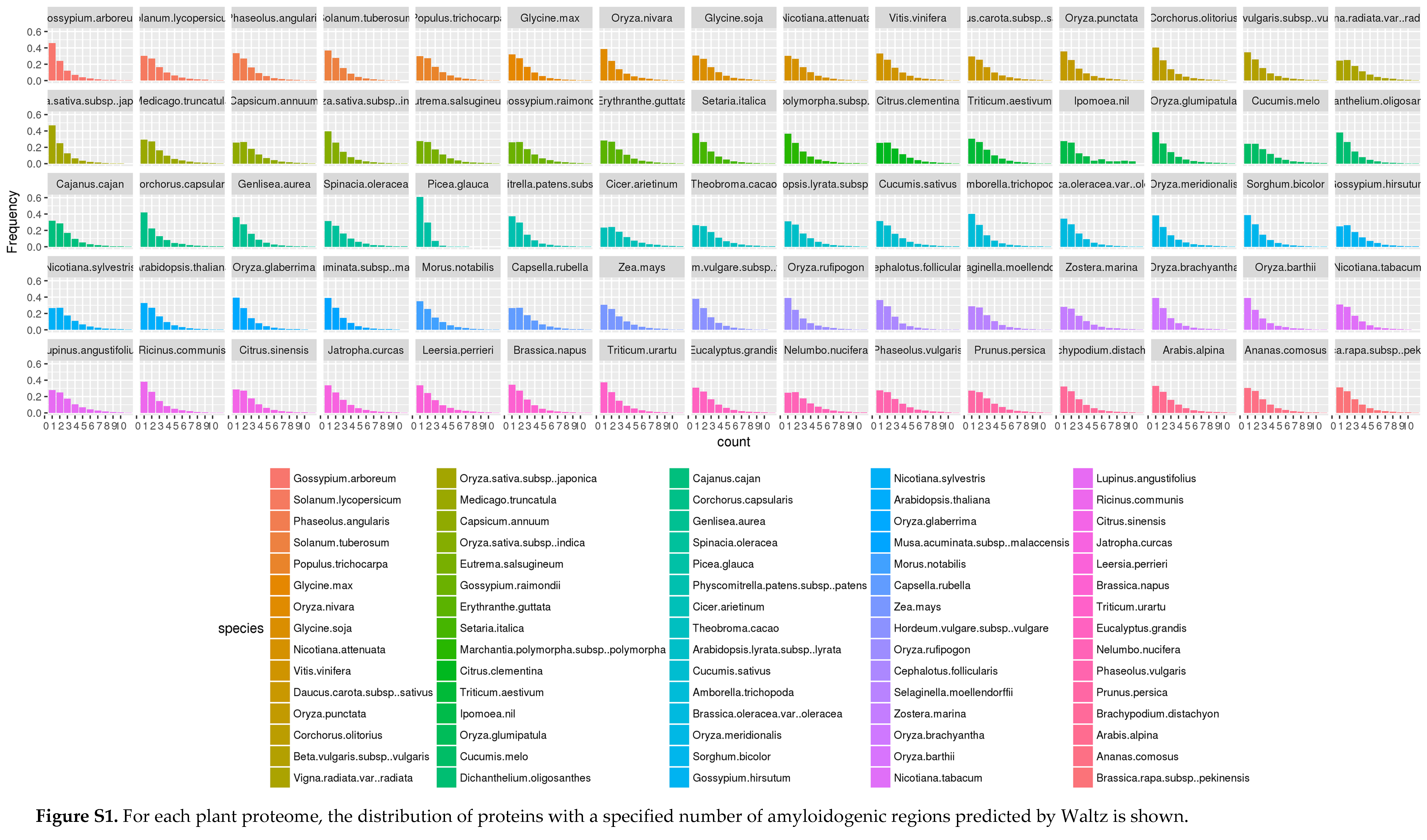

Supplement: Supplementary file 1 [file ijms-18-02155-s001.zip › Fig S1.tif]

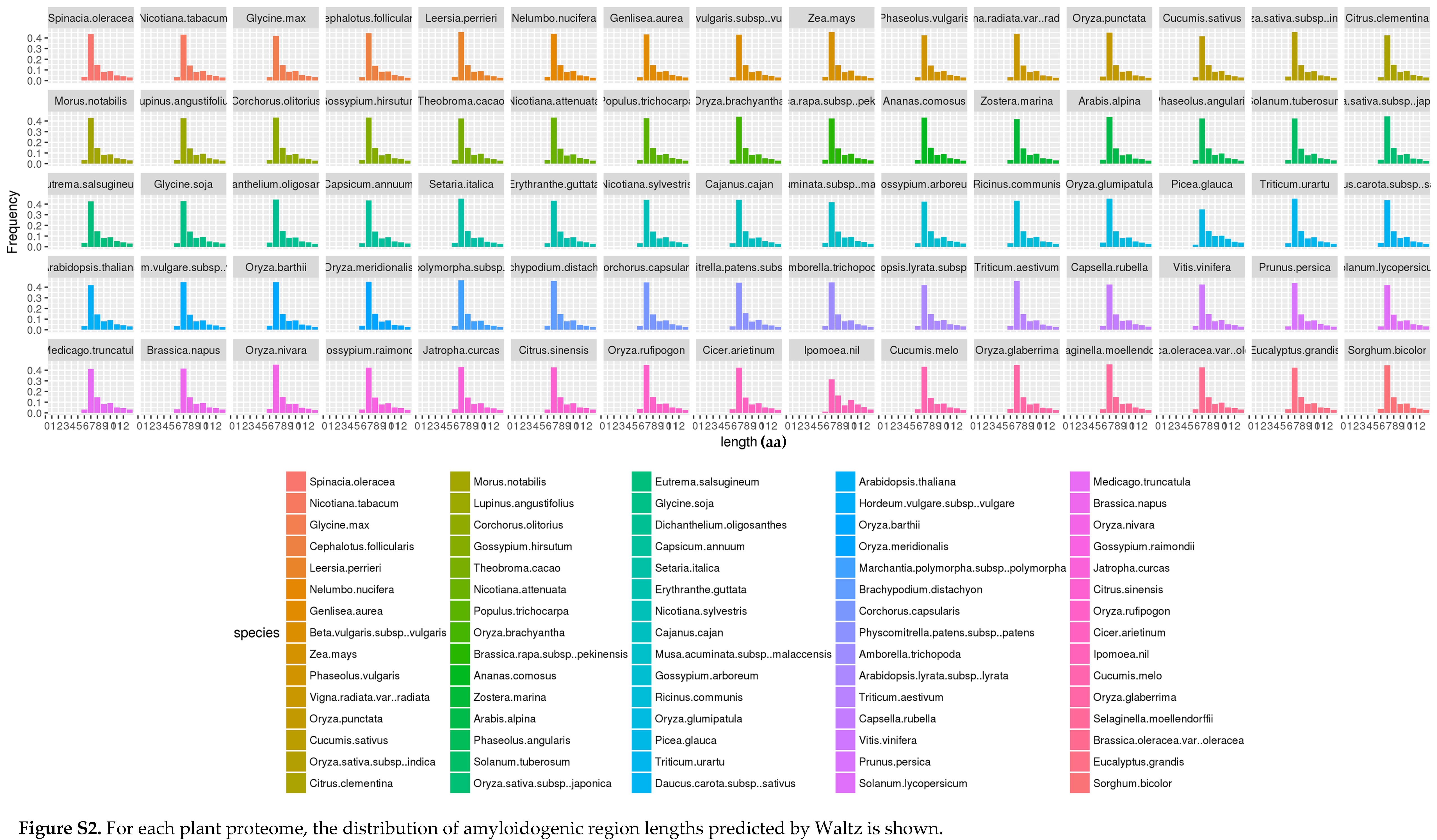

Supplement: Supplementary file 1 [file ijms-18-02155-s001.zip › Fig S2.tif]

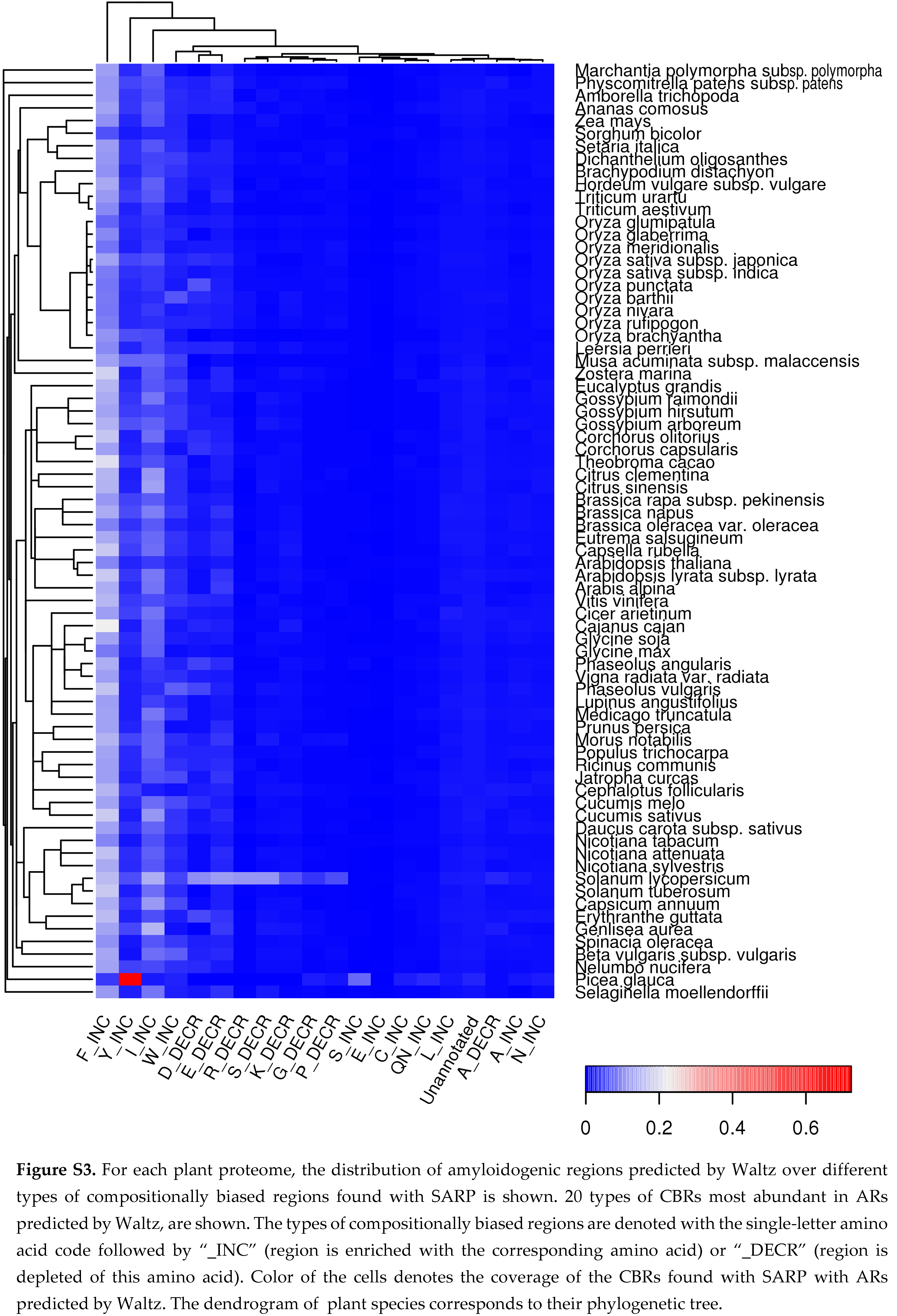

Supplement: Supplementary file 1 [file ijms-18-02155-s001.zip › Fig S3.tif]

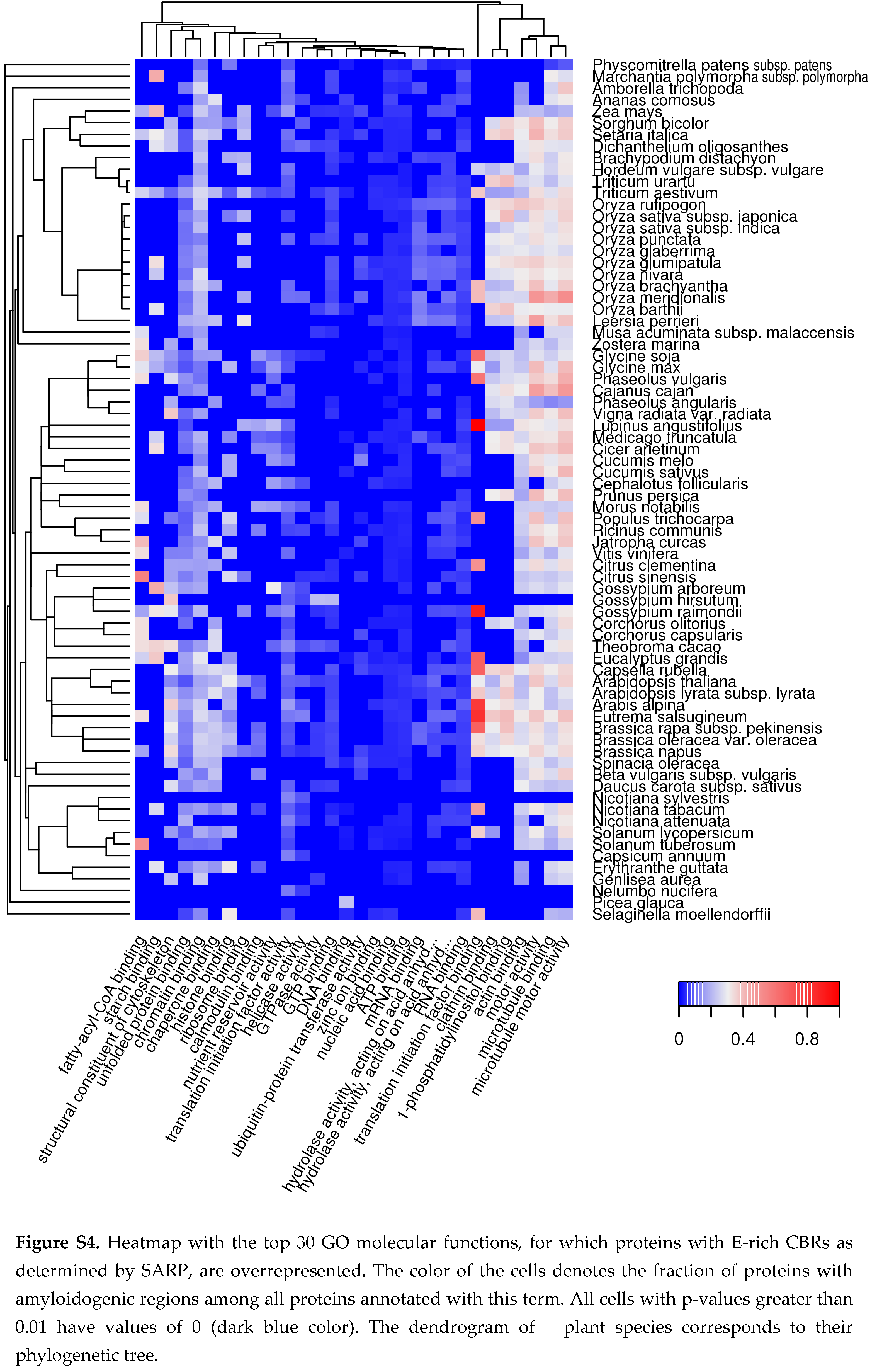

Supplement: Supplementary file 1 [file ijms-18-02155-s001.zip › Fig S4.tif]

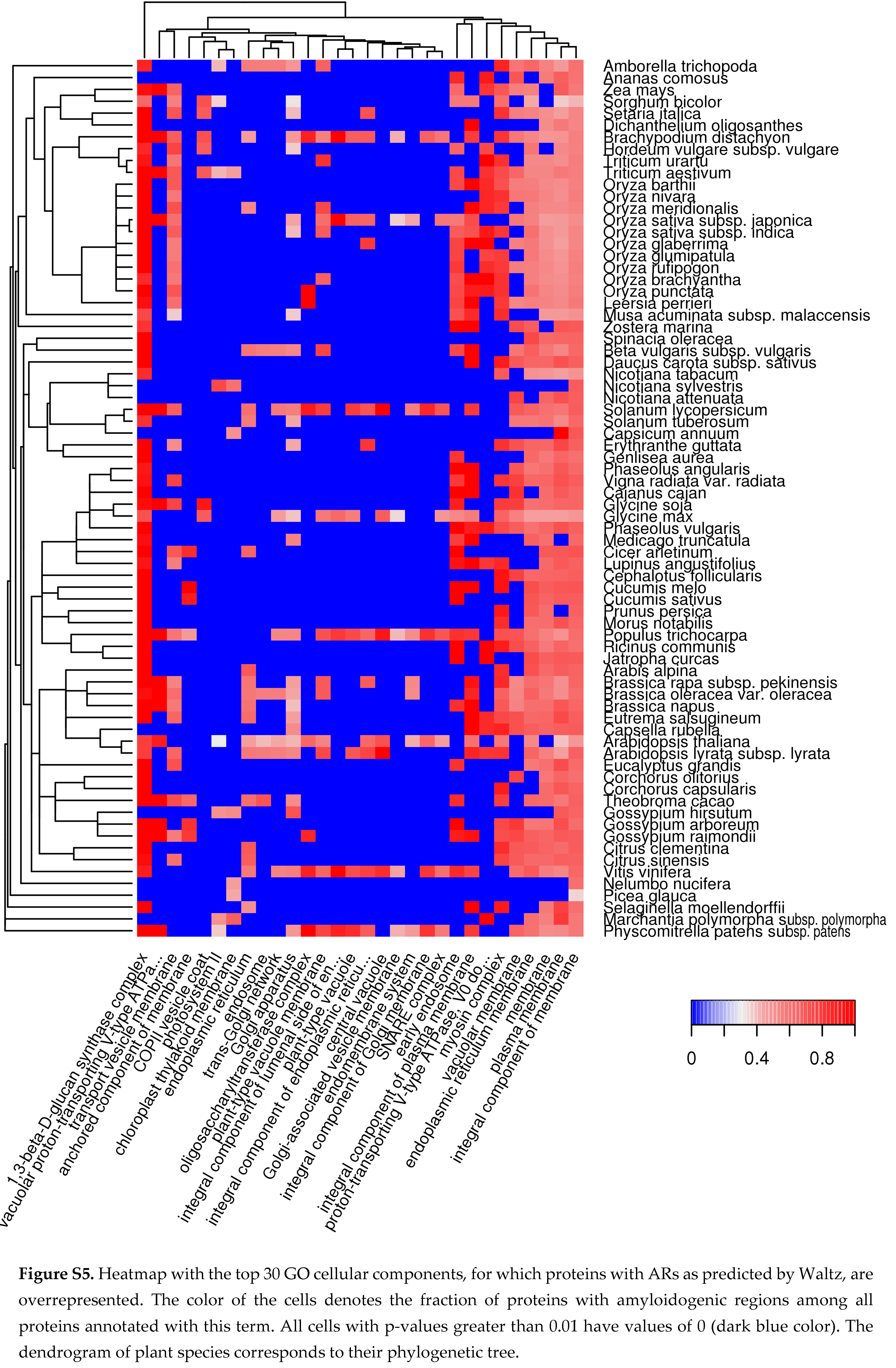

Supplement: Supplementary file 1 [file ijms-18-02155-s001.zip › Fig S5.tif]

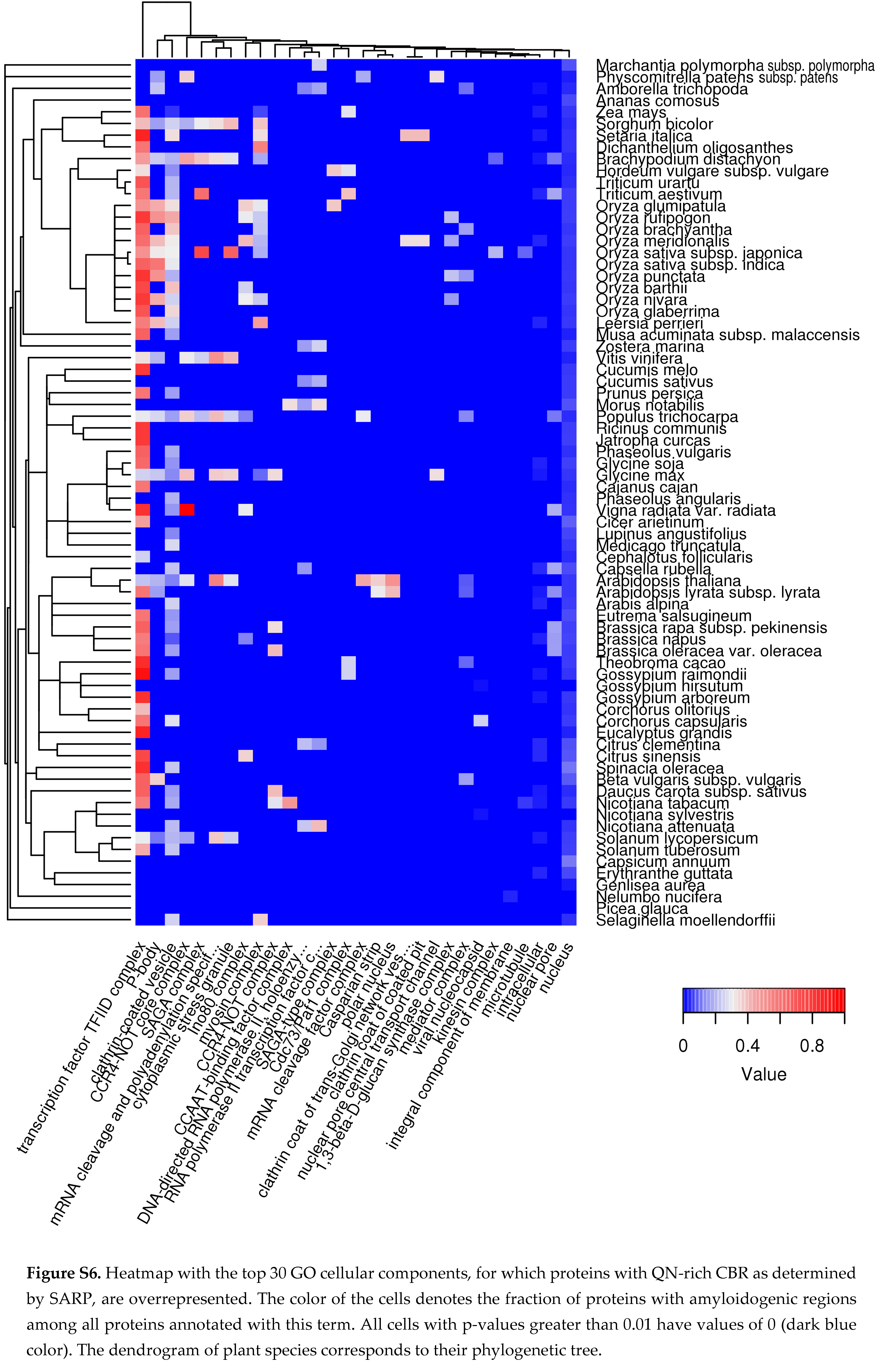

Supplement: Supplementary file 1 [file ijms-18-02155-s001.zip › Fig S6.tif]

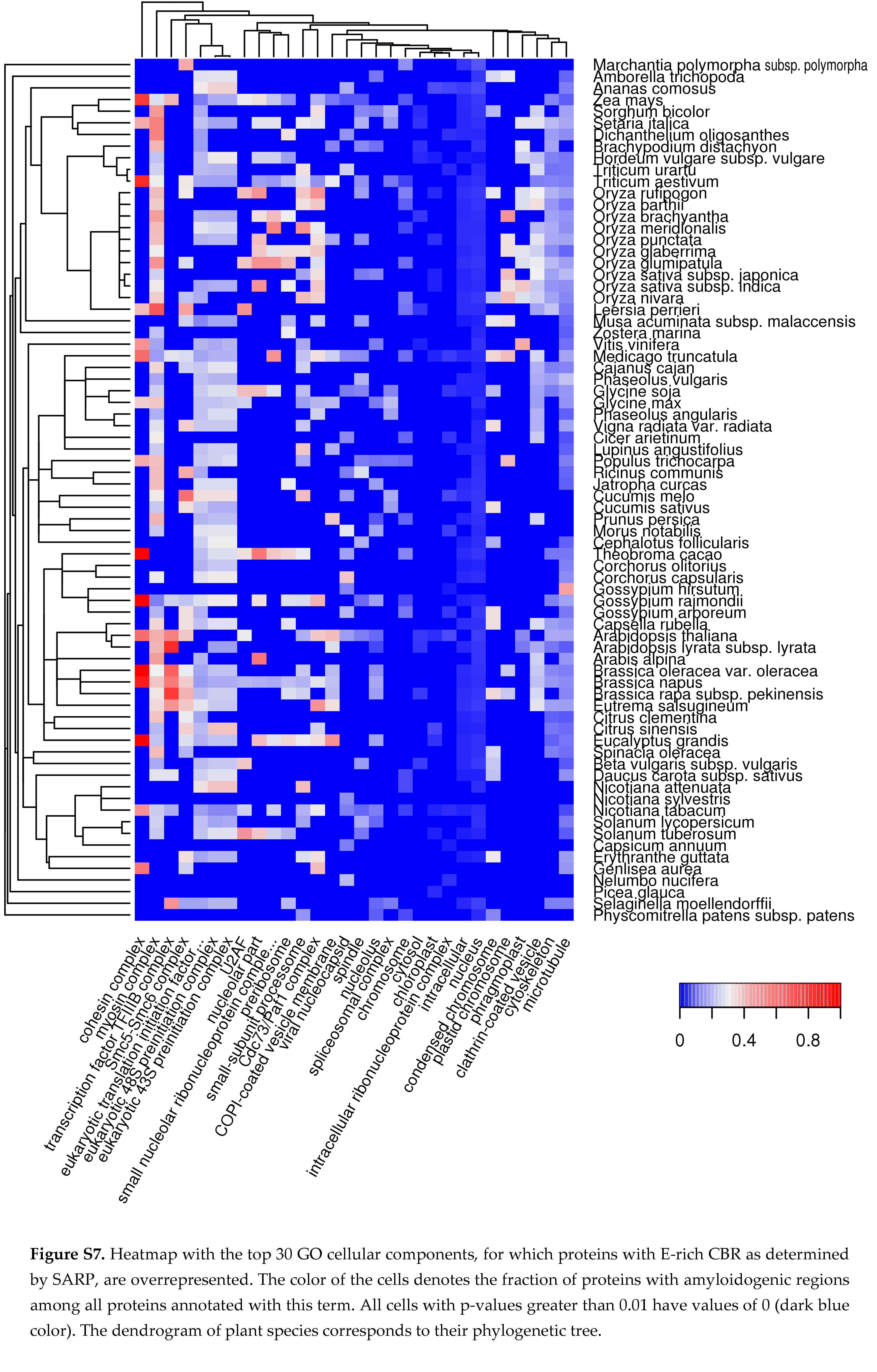

Supplement: Supplementary file 1 [file ijms-18-02155-s001.zip › Fig S7.tif]

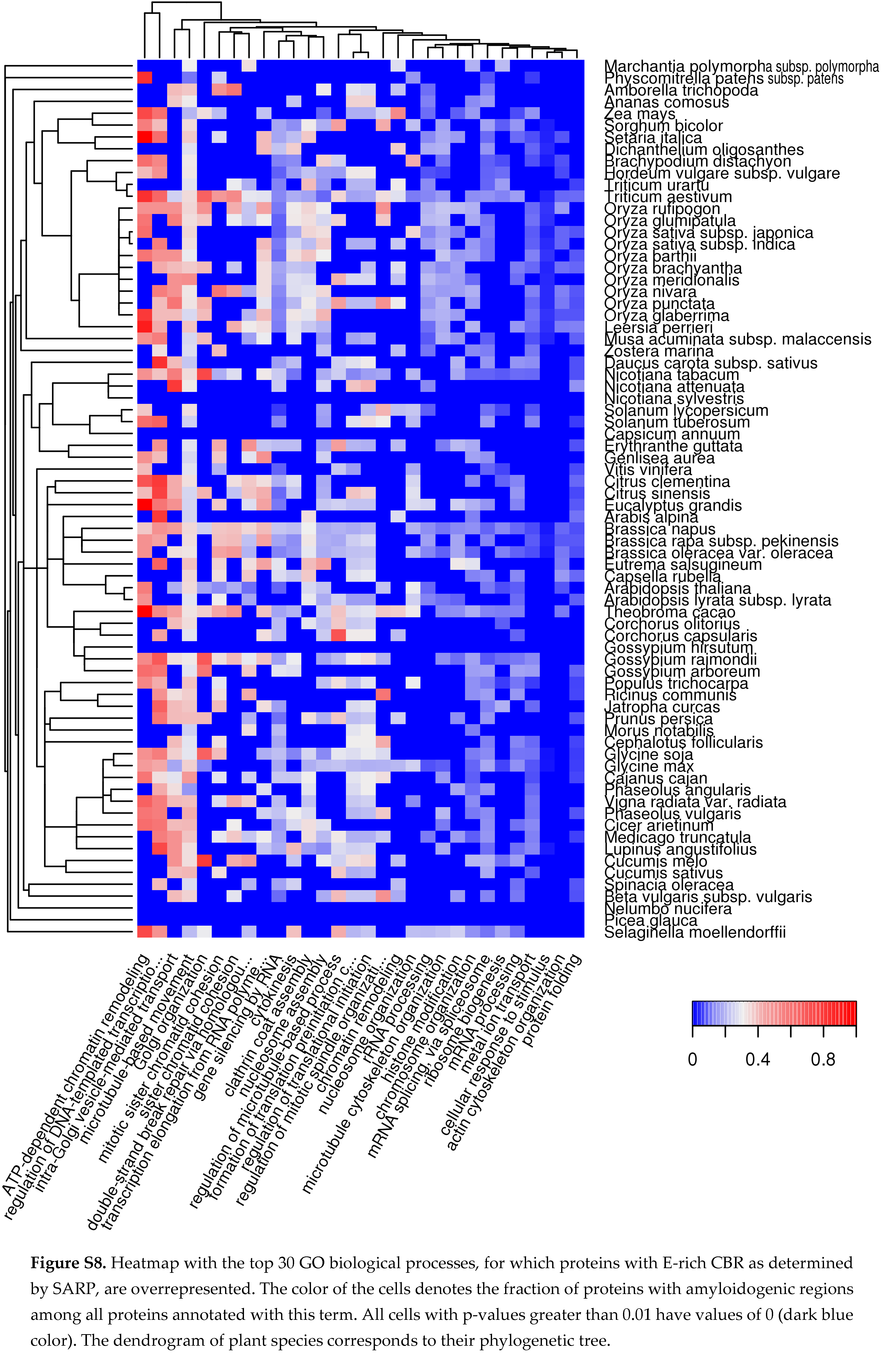

Supplement: Supplementary file 1 [file ijms-18-02155-s001.zip › Fig S8.tif]

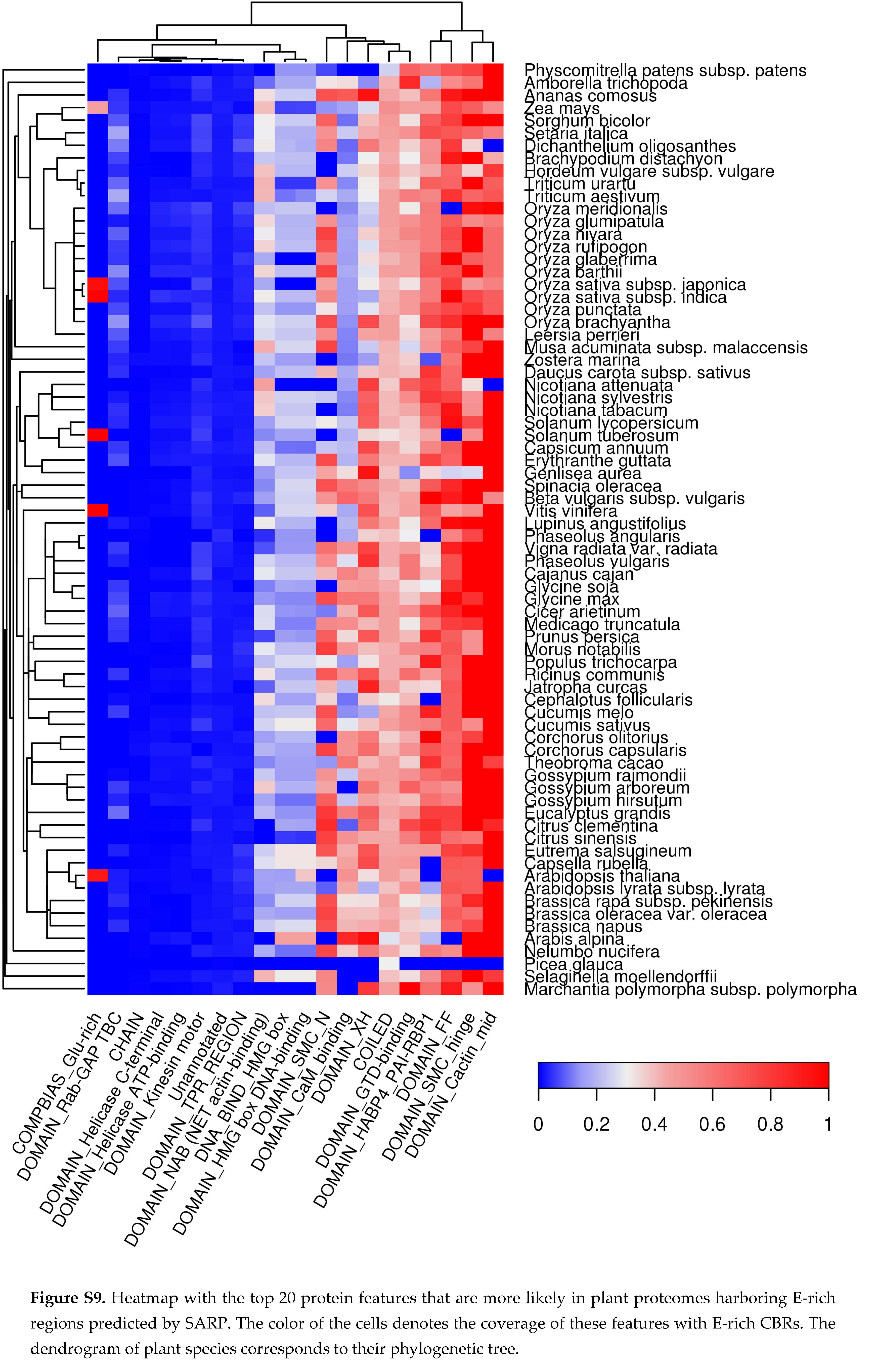

Supplement: Supplementary file 1 [file ijms-18-02155-s001.zip › Fig S9.tif]
